# Supplementary material for: Effects of a school-based intervention on 24-hour movement behaviors in adolescents: A quasi-experimental study
Source: PLoS One. 2025 Nov 21;20(11):e0336696. doi: 10.1371/journal.pone.0336696 (PMC12637913; doi:10.1371/journal.pone.0336696)
Supplement: Attachment — Submitted filename: Review.pdf [file pone.0336696.s002.pdf]

Effects of a school-based intervention on 24-hour movement behaviors in adolescents: A quasi-experimental study

I would like to sincerely thank the authors for the thorough and careful work carried out in addressing the reviewers' comments. The manuscript entitled "Effects of a school-based intervention on 24-hour movement behaviors in adolescents: A quasi-experimental study" represents a valuable contribution to the scientific literature, particularly in the field of adolescent health promotion and educational interventions.

Abstract

The summary clearly presents the objectives, design, and main results, highlighting the effects on sleep and screen time, as well as some improvements in motivational constructs. However, the effects of the intervention are not shown. I suggest adding the main results of your study.

Furthermore, it would be advisable to highlight the practical value of these partial improvements and conclude with a more explicit projection towards future research and the applicability of these interventions in school contexts.

Introduction

The theoretical framework is well-founded and structured. It presents the current state of the art on the subject under study and justifies the need to address the problem.

However, there are a few points I would like to highlight in this section that would be useful for improving the understanding of this heading. First, the authors should improve the connection between paragraphs. There are too many jumps between ideas from one paragraph to another, and the explanations are very brief. It would be necessary to expand this section a little more.

In addition, it would be interesting to include a brief paragraph on compliance with these recommendations, when they were established, and what those guidelines are. Here, the study by Tremblay et al. (2016) should be mentioned. Next, in this same paragraph, the authors should include a paragraph on the prevalence of compliance with the 24-hour recommendations for each of these behaviours. This would further reinforce the need to address this problem. For this paragraph, they could rely on the citation from Tapia-Serrano et al. (2022). Here are both references:

Tremblay, M. S., Carson, V., Chaput, J. P., Connor Gorber, S., Dinh, T., Duggan, M., ... & Zehr, L. (2016). Canadian 24-hour movement guidelines for children and youth: an integration of physical activity, sedentary behaviour, and sleep. *Applied physiology, nutrition, and metabolism*, *41*(6), S311-S327.

Tapia-Serrano, M. A., Sevil-Serrano, J., Sanchez-Miguel, P. A., Lopez-Gil, J. F., Tremblay, M. S., & Garcia-Hermoso, A. (2022). Prevalence of meeting 24-Hour Movement Guidelines from pre-school to adolescence: A systematic review and meta-analysis including 387,437 participants and 23 countries. *Journal of sport and health science*, *11*(4), 427-437.

In addition, I have found that some important references are missing from the introduction, which I will detail below.

The reference to Rollo et al. (2020) is important to include in the first paragraph, where they point out the effects of the 24-hour recommendations on young people's health, as this review has a specific section to clarify these findings. I am attaching the reference:

Rollo, S., Antsygina, O., & Tremblay, M. S. (2020). The whole day matters: understanding 24-hour movement guideline adherence and relationships with health indicators across the lifespan. *Journal of sport and health science*, *9*(6), 493-510.

It is important to mention the reference to Rodrigo-Sanqoquín in the section on the effect of school interventions based on the 24-HMB, as it is the only review that analyses the effect of these school interventions. I am attaching the reference:

Rodrigo-Sanjoaquín, J., Tapia-Serrano, M. Á., López-Gil, J. F., & Sevil-Serrano, J. (2025). Effects of school-based interventions on all 24-hour movement behaviours in young people: a systematic review and meta-analysis of randomised controlled trials. *BMJ Open Sport & Exercise Medicine*, *11*(2).

The section on SDT is very brief. I do not fully understand the reasons why you decided to propose this intervention under the postulates of this theoretical framework. There are studies that suggest that interventions based on theoretical frameworks are more effective, but these reviews are not cited in your document. Please explain this postulate better and add the citations on which you base this claim.

Finally, the state of the art on the effect of 24-HMB behaviours is unclear. Just before presenting the objectives and hypotheses, the authors should add a paragraph showing previous interventions in adolescents and based on those results, add what led them to consider this study. In other words, why are they analysing the effect of 24-HMB on quality of life and not on other variables? I imagine there is previous evidence on which they based their decision. This would reinforce the idea behind their approach and justify their intervention; otherwise, these are very general and vague ideas.

Method

The authors state: 'It was conducted over 24 weeks (12 weeks of intervention and 12 weeks of follow-up) in two schools located in Bello, Colombia.' This sentence is incorrect. Their intervention lasts 12 weeks, which is the time during which they are intervening. The other 12 weeks are a follow-up period, but this does not count towards the duration of the intervention. Modify this and revise the rest of the document accordingly.

Review and improve the section on instruments. It is not correct to include the KIDSCREEN questionnaire and the motivation questionnaire in the same paragraph. Ideally, you should add a paragraph for each variable and instrument.

Expand the information in this section. You must add the factor loadings and Cronbach's alpha for each instrument to make it correct. Also add how the factors for each instrument are formed. I suggest you look at some sample articles for support and improve this section. It is very poor and unscientific.

Did you also fill out the questionnaires? What actions and strategies were implemented? Once the intervention was completed, were they offered the opportunity to carry out the intervention so that they could benefit from the programme? Please add this information to the intervention section.

The statistical analysis section is solid but could be improved in several ways. First, although it is indicated that a significance threshold of $p < .05$ was used, the effect sizes are not reported. This element is essential for assessing the practical magnitude of the findings and not just their statistical significance. Including measures such as Cohen's d, partial η^2, or the marginal and conditional R^2 values of the mixed linear models would provide a much more complete context for interpreting the results.

On the other hand, when analysing multiple variables related to sleep, physical activity, screen time, ADHD constructs and quality of life, the risk of type I error increases. However, it is not specified whether any correction method for multiple comparisons, such as Bonferroni, Holm or FDR, was applied. Making this aspect explicit would reinforce the robustness of the analysis.

It would also be advisable to justify in greater detail the use of parametric and non-parametric tests. The text states that both were used for baseline comparisons but does not clarify which variables were analysed with each test or under what criteria (such as normality of data or homogeneity of variances). Clarification in this regard would provide greater methodological transparency.

Please address these issues in this section. I suggest that you support and/or revise this section based on some previous intervention studies, as it is too brief and leaves many questions unanswered for readers.

Results

Looking at the results for the screen time variable, we can see that this variable has been overestimated. Although the questionnaire used to measure screen time is valid and reliable, its calculation of the resulting average time is excessive and unrealistic, which could affect the accuracy of the results.

For example, the authors report that the total screen time of the participants in the experimental group (which can also be extended to the control group) is 7.51 hours/day (Table 3). This seems excessive and unrealistic for children and adolescents, suggesting a possible overestimation by the participants. It is suggested that the maximum values be reviewed to identify possible discrepancies that could bias the results.

Discussion

The discussion contains some redundancies, especially in the explanation of the results related to moderate to vigorous physical activity and screen time, which could be summarised to improve the flow of the text. In some passages, the tone tends to overestimate the effectiveness of the intervention, so it would be more appropriate to qualify the language and refer to partial or limited effects, so that the results are aligned with the actual evidence. The absence of effects on health-related quality of life is mentioned briefly, but it would be useful to discuss in greater depth whether this was due to limitations in the design, the measurement instrument used, or the short duration of the

intervention. It would also be advisable to reflect further on the role of school and family contexts in the sustainability of the changes observed in 24-hour behaviours, as their influence can be decisive in maintaining long-term effects. Finally, the section could be enriched with a more practical analysis of the findings, highlighting that reductions of between 1.5 and 2 hours per day in screen time are clinically relevant for adolescent health, even if the recommendation of two hours or less per day is not fully achieved.

Finally, the section on limitations, strengths and suggestions for future studies should be restructured under the same heading.

Conclusion

The conclusion summarises the findings well, highlighting improvements in sleep, reduced screen time and positive effects on some motivational constructs. However, the interpretation is limited, as it does not clarify that the effects were partial and that there were no significant improvements in physical activity or quality of life. It would be advisable to emphasise the practical value of modest changes in sleep and screen use, as well as to conclude with a clearer projection towards future research and application in school contexts.

References.

Please review the references section as they appear to be in a different font.

Also review Figure 1, as it is of poor quality.
