# Supplementary material for: Effects of a school-based intervention on 24-hour movement behaviors in adolescents: A quasi-experimental study
Source: PLoS One. 2025 Nov 21;20(11):e0336696. doi: 10.1371/journal.pone.0336696 (PMC12637913; doi:10.1371/journal.pone.0336696)
Supplement: Attachment — Submitted filename: Review 2.pdf [file pone.0336696.s004.pdf]

Effects of a school-based intervention on 24-hour movement behaviors in adolescents: A quasi-experimental study

Firstly, I would like to thank the editor for the opportunity to review your manuscript entitled ‘Effects of a school-based intervention on 24-hour movement behaviors in adolescents: A quasi-experimental study’. I appreciate the opportunity to review your work, which addresses a topic of great relevance to public health and adolescent well-being. The study has the potential to make an important contribution in the field of school intervention and the promotion of healthy habits.

Below, I present my comments and suggestions, which I hope will contribute to improving the quality and clarity of the article.

Strengths of the manuscript

The study is well designed, with an appropriate methodological structure that allows for rigorous evaluation of the effects of the school intervention. The use of Self-Determination Theory (SDT) as a theoretical framework is appropriate, and the quasi-experimental approach with control and experimental groups provides a solid basis for the analyses presented. Furthermore, the topic of 24-hour movement behaviours (physical activity, sedentary behaviour, and sleep) is highly topical and relevant, given the growing interest in the integration of these three aspects in adolescent health.

Minor comments

Despite the strengths mentioned above, there are several aspects that could benefit from further revision to improve the clarity and depth of the analysis. Below are some suggestions and observations:

Clarification on support and control strategies in SDT

One of the most relevant points in this study is the use of SDT-based strategies. However, I would like to suggest clarifying whether the autonomy, competence, and relatedness strategies implemented in the intervention are primarily aimed at supporting these psychological needs or controlling them. According to SDT, these needs can be met in ways that either support or control individuals' motivation, and it is important to highlight this point in the article, as it could have implications for the interpretation of the results. This distinction is fundamental to understanding how the intervention impacts participants' motivation and behaviours.

Updating the tables

In Table 2 and other related tables, I suggest that the information be updated to better reflect the intervention strategies in relation to SDT. Specifically, it should be indicated whether the strategies applied are designed to promote an approach that supports psychological needs or whether there is any form of control over them. Including this information in the tables will provide greater clarity about the intervention design and its alignment with the proposed theory.

While I appreciate the authors' response to the reviewers' comments, I must point out that, for future occasions, it would be advisable to send a supplementary response to the reviewers' comments. The format in which the authors have responded to the comments makes it difficult to review the article, as the changes made to the manuscript are not clearly specified, nor are the exact sections that have been changed or added indicated. A more detailed and structured response would help reviewers to track the changes more efficiently, ensuring a smoother and more comprehensible review.

In short, the manuscript has great potential, but to ensure that the impact of the intervention and its alignment with SDT are clearly understood, I suggest incorporating the aforementioned modifications. I believe it is important to highlight whether the intervention strategies favour a supportive or controlling approach to psychological needs, as this distinction may influence the interpretation of the results and the applicability of the intervention to other contexts.
