# Supplementary material for: Effects of a school-based intervention on 24-hour movement behaviors in adolescents: A quasi-experimental study
Source: PLoS One. 2025 Nov 21;20(11):e0336696. doi: 10.1371/journal.pone.0336696 (PMC12637913; doi:10.1371/journal.pone.0336696)
Supplement: Attachment — Submitted filename: Point-by-point response.docx [file pone.0336696.s005.docx]

**Manuscript title: Effects of a school-based intervention on 24-hour movement behaviors in adolescents: A quasi-experimental study.**

Point-by-point response

| **Reviewer 1** | | | |
| --- | --- | --- | --- |
|  | ***Comment*** | ***Response*** | ***Place of Response*** |
| **#** | **Suggestions for improvement:** | | |
| 2 | The manuscript states that one school was the experimental group (EG) and another the control group (CG). This convenience allocation risks baseline differences and school-level confounding. Explicitly acknowledge this in the limitations and discuss how it may have affected internal validity. | We thank the reviewer for this valuable observation. We have revised the Limitations section of the manuscript to explicitly acknowledge this issue. | Lines  424-437. |
| 3 | Given the proximity and similarity of the schools, describe measures taken to avoid information spillover between groups. | Thanks for this comment. We added information to clarify this aspect. | Lines  130-133. |
| 4 | The sample size calculation is based on proportions from a prior study but does not account for clustering at the school level, which likely reduces effective sample size. This should be noted as a limitation. | Thanks for noticing it. We have included it as a limitation. | Lines  426-429. |
| 5 | All behavioral measures (MVPA, sleep, screen time) were self-reported, which can over- or under-estimate true values. This should be more explicitly acknowledged in the limitations, with discussion of potential implications. | Thanks for this suggestion. We have expanded this limitation. | Lines  430-433. |
| 6 | While you cite validation studies, specify whether these instruments were culturally adapted/validated for Colombian youth or Spanish-speaking populations in Latin America. | We thank the reviewer for this valuable comment. We have clarified that the instruments used were transculturally adapted and validated in Spanish-speaking populations in Latin America, including Colombian youth. | Lines  160-164, 167-169, 171-172, 178-179. |
| 7 | The lack of accelerometer-based data is a key limitation, particularly for detecting changes in physical activity. | We have articulated this comment in response to suggestion #5. | Lines  430-433. |
| 8 | While p-values are provided, effect sizes (e.g., Cohen’s d, partial η², or odds ratios with 95% CIs) should be reported for main outcomes to contextualize practical significance. | Thanks for this helpful suggestion. We have used mixed-effects models, and for this, measures such as Cohen’s d or partial η² are not always appropriate. Instead, we reported the conditional R², which reflects the variance explained by both fixed and random effects in the mixed models, and the Relative Risk (RR), to provide a more direct and interpretable measure of association.  We have revised the Data analysis section to clarify our rationale for using these measures | Lines  239-242. |
| 9 | Given the large number of statistical tests, discuss whether adjustments (e.g., Bonferroni, FDR) were considered to control Type I error. | We thank the reviewer for raising this point. We have added it as a limitation. | Lines  242-243. |
| 10 | The discussion sometimes implies meaningful trends where p-values are >.05. These should be interpreted cautiously, avoiding overstatement. | Thanks for this suggestion. We have edited the information to make it clearer. | Lines  375-376. |
| 11 | The intervention is detailed in Table 1, but its theoretical integration with SDT could be made more explicit. For example:  How did each activity target autonomy, competence, or relatedness? Please see a recent manuscript by Ahmadi et al., (2023) in which a classification system for autonomy, competence and relatedness support techniques was developed. I think this manuscript would benefit from insights from Ahmadi et al., (2023) paper.  Ahmadi, A., Noetel, M., Parker, P., Ryan, R. M., Ntoumanis, N., Reeve, J., Beauchamp, M., Dicke, T., Yeung, A., Ahmadi, M., Bartholomew, K., Chiu, T. K. F., Curran, T., Erturan, G., Flunger, B., Frederick, C., Froiland, J. M., González-Cutre, D., Haerens, L., . . . Lonsdale, C. (2023). A classification system for teachers’ motivational behaviors recommended in self-determination theory interventions. Journal of Educational Psychology, 115(8), 1158–1176. <https://doi.org/10.1037/edu0000783> | We thank the reviewer for these valuable observations. We have added a table detailing the intervention strategies implemented, thereby strengthening the scientific rigor of this section. | Lines  210-213. |
| 12 | Were teachers trained in autonomy-supportive behaviors, and if so, how was fidelity monitored? | We thank the reviewer for this comment. Fidelity was ensured through self-monitor and treatment manual. We have added this information to the paragraph where the intervention is described. | Lines  199-203. |
| 13 | Consider summarizing the four components more concisely in the main text and moving full weekly details to supplementary material. | We think that it is important to present this description in the main document as a key source of information for reproducibility. | Lines  210-213. |
| 14 | The discussion could better link observed behavioral changes (e.g., in screen time and sleep) to the specific intervention strategies and SDT constructs.  While possible explanations are given (short duration, lack of extracurricular sports), consider also whether barriers in the school or community context were at play. | Thanks for this suggestion. We have expanded the discussion to address these topics. | Lines  378-381, 408-415. |
| 15 | Deepen the contrast between your findings and similar interventions in different sociocultural contexts. | Thanks for this suggestion. We have expanded the discussion to address the topic. | Lines  410-415. |
| 16 | Add a brief paragraph on how findings could inform school health policies in Colombia or Latin America. | Thanks for this suggestion. We have expanded the discussion to address the topic. | Lines  416-421. |

|  | **Reviewer 2** | | |
| --- | --- | --- | --- |
|  | ***Comment*** | ***Response*** | ***Page of Response*** |
| 17 | Below are my suggestions and criticisms:  In the abstract and introduction, a description and definition of Self-Determination Theory is needed. | Thanks for this suggestion. Due to word limitations in the abstract, we only expanded the description and definition of Self-Determination Theory in the introduction. | Lines  88-99. |
| 18 | In the abstract, quantitative results should be included in the abstract. | Thanks for this suggestion. We included some quantitative results in the abstract. | Lines  43-46. |
| 19 | In the introduction, a description of the public health burden is needed. For example, the prevalence of adolescents who meet physical activity, sedentary behavior, and sleep recommendations. | We appreciate the suggestion. We included a paragraph on the prevalence of compliance with the 24-hour recommendations for each of these behaviors. | Lines  67-78. |
|  | **Methods** | | |
| 20 | What minimum difference in outcomes is the sample size/power able to detect? | Thanks for your question. Part of this information is still included in the manuscript. We only added this sentence:  *“Therefore, the minimum difference to be detected in compliance was 48% in favor of the experimental group.”* | Lines 138-139. |
| 21 | Why couldn’t the authors utilize a randomized control trial? | Thank you for this important comment. We acknowledge that randomized controlled trials are the gold standard; however, conducting one in our school context was not feasible. Random allocation of students or classrooms would have disrupted existing structures and routines, and resource limitations prevented implementation of a full RCT. Therefore, consistent with research on quasi-experimental approaches in naturalistic settings, we adopted a quasi-experimental design to evaluate the intervention under real-world conditions while maintaining ecological validity. We also explicitly acknowledge the inherent limitations of this design compared to randomized trials.  Reference:  Bärnighausen T, Tugwell P, Røttingen JA, Shemilt I, Rockers P, Geldsetzer P, et al. Quasi-experimental study designs series—paper 4: uses and value. J Clin Epidemiol. 2017 Sep;89:21–29. doi:10.1016/j.jclinepi.2017.03.012. | Lines 424-426. |
| 22 | More of the details of the validity and the reliability of each of the 24-Hour Movement Behaviors. | Thanks for your comment. We added more information about 24-Hour Movement Behaviors questionnaire. | Lines 160-164, 167-169, 171-172, 178-179. |
| 23 | For the intervention, how was Self-Determination Theory used to develop the intervention? | Thanks for your comment. We added one table to complete this request:  Table 2. Behavior Change Techniques and Strategies Based on SDT Constructs Implemented in the School-Based Intervention Program. | Lines 210-213. |
|  | **Results** | | |
| 24 | At the top of page 11, lines 185 to 188, could the authors describe the direction in the measure of association? | Thanks for this suggestion. We described the direction in the measure of association of the sleep time. | Lines 267-270. |
|  | **Discussion** | | |
| 25 | The Discussion often repeats the findings described in the results section. It would be helpful if the authors could describe how the findings not only fit in with other studies but also explain any public health implications. | Thanks for this suggestion. We have expanded the discussion to address the implications of findings on school health policies in Colombia. | Lines 366-374, 416-421. |
| 26 | Could the authors explain the limitations in the context of the current study. How did the quasi-experimental design affect internal validity, use of self-report questionnaires as being prone to self-report bias. | Thanks for this suggestion. We have expanded these limitations. | Lines 424-437. |

|  | **Reviewer 3** | | |
| --- | --- | --- | --- |
|  | ***Comment*** | ***Response*** | ***Page of Response*** |
|  | **Abstract** | | |
| 27 | The summary clearly presents the objectives, design, and main results, highlighting the effects on sleep and screen time, as well as some improvements in motivational constructs. However, the effects of the intervention are not shown. I suggest adding the main results of your study. | Thanks for this suggestion. We included some quantitative results in the abstract. | Lines  43-46. |
| 28 | Furthermore, it would be advisable to highlight the practical value of these partial improvements and conclude with a more explicit projection towards future research and the applicability of these interventions in school contexts. | Thanks for your recommendation. We added a sentence explaining future research and the applicability of these school-based interventions. | Lines  50-53. |
|  | **Introduction** | | |
| 29 | The theoretical framework is well-founded and structured. It presents the current state of the art on the subject under study and justifies the need to address the problem.  However, there are a few points I would like to highlight in this section that would be useful for improving the understanding of this heading. First, the authors should improve the connection between paragraphs. There are too many jumps between ideas from one paragraph to another, and the explanations are very brief. It would be necessary to expand this section a little more. | We sincerely appreciate the reviewer’s positive assessment of the theoretical framework and the constructive suggestions provided. We acknowledge the need to strengthen the coherence between paragraphs and to expand some explanations in order to improve the clarity and depth of this section. Accordingly, we have revised the text to ensure smoother transitions between ideas and have incorporated additional details to reinforce the arguments and enhance the overall comprehension of the framework. | Lines  67-78, 88-117. |
| 30 | In addition, it would be interesting to include a brief paragraph on compliance with these recommendations, when they were established, and what those guidelines are. Here, the study by Tremblay et al. (2016) should be mentioned. Next, in this same paragraph, the authors should include a paragraph on the prevalence of compliance with the 24-hour recommendations for each of these behaviours. This would further reinforce the need to address this problem. For this paragraph, they could rely on the citation from Tapia-Serrano et al. (2022). Here are both references:  Tremblay, M. S., Carson, V., Chaput, J. P., Connor Gorber, S., Dinh, T., Duggan, M., ... & Zehr, L. (2016). Canadian 24-hour movement guidelines for children and youth: an integration of physical activity, sedentary behaviour, and sleep. Applied physiology, nutrition, and metabolism, 41(6), S311-S327.  Tapia-Serrano, M. A., Sevil-Serrano, J., Sanchez-Miguel, P. A., Lopez-Gil, J. F., Tremblay, M. S., & Garcia-Hermoso, A. (2022). Prevalence of meeting 24-Hour Movement Guidelines from pre-school to adolescence: A systematic review and meta-analysis including 387,437 participants and 23 countries. Journal of sport and health science, 11(4), 427-437. | We appreciate the suggestion and have added a paragraph including Tremblay et al. (2016) and Tapia-Serrano et al. (2022) to reinforce the justification of the problem. | Lines  67-78. |
| 31 | In addition, I have found that some important references are missing from the introduction, which I will detail below.  The reference to Rollo et al. (2020) is important to include in the first paragraph, where they point out the effects of the 24-hour recommendations on young people's health, as this review has a specific section to clarify these findings. I am attaching the reference:  Rollo, S., Antsygina, O., & Tremblay, M. S. (2020). The whole day matters: understanding 24-hour movement guideline adherence and relationships with health indicators across the lifespan. Journal of sport and health science, 9(6), 493-510. | We appreciate the reviewer’s observation. Accordingly, we have incorporated the reference by Rollo et al. (2020) in the introduction to strengthen the section and clarify the health effects associated with adherence to the 24-hour movement guidelines. | Line  79-81, 100-117. |
| 32 | It is important to mention the reference to Rodrigo-Sanjoquín in the section on the effect of school interventions based on the 24-HMB, as it is the only review that analyses the effect of these school interventions. I am attaching the reference:  Rodrigo-Sanjoaquín, J., Tapia-Serrano, M. Á., López-Gil, J. F., & Sevil-Serrano, J. (2025). Effects of school-based interventions on all 24-hour movement behaviours in young people: a systematic review and meta-analysis of randomised controlled trials. BMJ Open Sport & Exercise Medicine, 11(2). | We appreciate the reviewer’s suggestion. In response, we have included the reference by Rodrigo-Sanjoaquín et al. (2025) in the section addressing school-based interventions, thereby strengthening the discussion on their effects on 24-hour movement behaviors. | Line  81-82. |
| 33 | The section on SDT is very brief. I do not fully understand the reasons why you decided to propose this intervention under the postulates of this theoretical framework. There are studies that suggest that interventions based on theoretical frameworks are more effective, but these reviews are not cited in your document. Please explain this postulate better and add the citations on which you base this claim. | We thank the reviewer for this observation. We have expanded the section on Self-Determination Theory to clarify the rationale for its use in the proposed intervention and have incorporated the corresponding references supporting the effectiveness of theory-based interventions. | Lines  88-99. |
| 34 | Finally, the state of the art on the effect of 24-HMB behaviours is unclear. Just before presenting the objectives and hypotheses, the authors should add a paragraph showing previous interventions in adolescents and based on those results, add what led them to consider this study. In other words, why are they analysing the effect of 24-HMB on quality of life and not on other variables? I imagine there is previous evidence on which they based their decision. This would reinforce the idea behind their approach and justify their intervention; otherwise, these are very general and vague ideas. | We appreciate the reviewer’s insightful comment. Accordingly, we have added a paragraph summarizing previous interventions in adolescents and clarified the rationale for analysing the effect of 24-HMB on quality of life, thereby reinforcing and justifying the proposed approach. | Lines  100-109. |
|  | **Method** | | |
| 35 | The authors state: ‘It was conducted over 24 weeks (12 weeks of intervention and 12 weeks of follow-up) in two schools located in Bello, Colombia.’ This sentence is incorrect. Their intervention lasts 12 weeks, which is the time during which they are intervening. The other 12 weeks are a follow-up period, but this does not count towards the duration of the intervention. Modify this and revise the rest of the document accordingly. | We thank the reviewer for this clarification. We have corrected the paragraph to specify that the intervention lasted 12 weeks, followed by a 12-week follow-up period, and we have revised the rest of the document to ensure consistency with this adjustment. | Line 127. |
| 36 | Review and improve the section on instruments. It is not correct to include the KIDSCREEN questionnaire and the motivation questionnaire in the same paragraph. Ideally, you should add a paragraph for each variable and instrument. | We appreciate the reviewer’s suggestion. The section on instruments has been revised, and the KIDSCREEN questionnaire and the motivation questionnaire are now presented in separate paragraphs to improve clarity and accuracy. | Lines  170-172. |
| 37 | Expand the information in this section. You must add the factor loadings and Cronbach's alpha for each instrument to make it correct. Also add how the factors for each instrument are formed. I suggest you look at some sample articles for support and improve this section. It is very poor and unscientific.  Did you also fill out the questionnaires? What actions and strategies were implemented? Once the intervention was completed, were they offered the opportunity to carry out the intervention so that they could benefit from the programme? Please add this information to the intervention section. | We thank the reviewer for these valuable observations. We have expanded the section by including the factor loadings and Cronbach’s alpha for each instrument, as well as a description of how the factors are formed. In addition, we have added a table detailing the intervention strategies implemented, thereby strengthening the scientific rigor of this section. | Lines  160-164, 167-169, 171-172, 178-179, 211-213. |
| 38 | The statistical analysis section is solid but could be improved in several ways. First, although it is indicated that a significance threshold of p < .05 was used, the effect sizes are not reported. This element is essential for assessing the practical magnitude of the findings and not just their statistical significance. Including measures such as Cohen's d, partial η², or the marginal and conditional R² values of the mixed linear models would provide a much more complete context for interpreting the results. | We appreciate the reviewer’s constructive comment. In response, we have reported the corresponding effect sizes (marginal/conditional R² values for the mixed linear models) to provide a more comprehensive interpretation of the results beyond statistical significance. | Lines  239-242. |
| 39 | On the other hand, when analyzing multiple variables related to sleep, physical activity, screen time, ADHD constructs and quality of life, the risk of type I error increases. However, it is not specified whether any correction method for multiple comparisons, such as Bonferroni, Holm or FDR, was applied. Making this aspect explicit would reinforce the robustness of the analysis. | We appreciate the reviewer’s observation. We have revised the paragraph to explicitly state that no adjustment or correction methods for multiple comparisons were applied, thereby clarifying this aspect of the statistical analysis. | Lines  242-243. |
| 40 | It would also be advisable to justify in greater detail the use of parametric and non-parametric tests. The text states that both were used for baseline comparisons but does not clarify which variables were analyzed with each test or under what criteria (such as normality of data or homogeneity of variances). Clarification in this regard would provide greater methodological transparency.  Please address these issues in this section. I suggest that you support and/or revise this section based on some previous intervention studies, as it is too brief and leaves many questions unanswered for readers. | We thank the reviewer for this important observation. We have revised the section to clarify the criteria used for applying parametric and non-parametric tests, specifying the variables analyzed with each approach and supporting these decisions with references from previous intervention studies to enhance methodological transparency. | Lines  228-234. |
|  | **Results** | | |
| 41 | Looking at the results for the screen time variable, we can see that this variable has been overestimated. Although the questionnaire used to measure screen time is valid and reliable, its calculation of the resulting average time is excessive and unrealistic, which could affect the accuracy of the results.  For example, the authors report that the total screen time of the participants in the experimental group (which can also be extended to the control group) is 7.51 hours/day (Table 3). This seems excessive and unrealistic for children and adolescents, suggesting a possible overestimation by the participants. It is suggested that the maximum values be reviewed to identify possible discrepancies that could bias the results. | We appreciate the careful observation of the screen time results. Following this comment, we reviewed the dataset and confirmed that the reported values are correct and consistent with the established scoring procedures for the questionnaire. While the average of 7.51 hours/day may seem high, it is consistent with the findings of recent studies in adolescent populations in Latin America and around the world, where average daily recreational screen time frequently exceeds 6–7 hours. These values reflect current trends in digital device use among adolescents and are particularly influenced by prolonged use during leisure periods, such as weekends. Therefore, while high, our results are representative of the study population and reinforce the importance of addressing screen time as a critical health behavior in this age group.  Zablotsky B, Arockiaraj B, Haile G, Ng AE. Daily screen time among teenagers: United States, July 2021–December 2023. NCHS Data Brief, no 513. Hyattsville, MD: National Center for Health Statistics. 2024. DOI: <https://dx.doi.org/10.15620/cdc/168509>  Fan H, Yan J, Yang Z, Liang K, Chen S. Cross-sectional associations between screen time and the selected lifestyle behaviors in adolescents. Front Public Health. 2022;10:932017. DOI: <https://dx.doi.org/10.3389/fpubh.2022.932017> . | Lines  288-290. |
|  | **Discussion** | | |
| 42 | The discussion contains some redundancies, especially in the explanation of the results related to moderate to vigorous physical activity and screen time, which could be summarised to improve the flow of the text. | Thank you for this comment. We've restructured the paragraph to avoid redundancies and clarify this point. | Lines  344-347. |
| 43 | In some passages, the tone tends to overestimate the effectiveness of the intervention, so it would be more appropriate to qualify the language and refer to partial or limited effects, so that the results are aligned with the actual evidence. | Thanks for this comment. We added information to clarify this aspect. | Lines 348. |
| 44 | The absence of effects on health-related quality of life is mentioned briefly, but it would be useful to discuss in greater depth whether this was due to limitations in the design, the measurement instrument used, or the short duration of the intervention. | Thanks for the comment. We've organized the paragraph and added a more in-depth explanation. | Lines  397-407. |
| 45 | It would also be advisable to reflect further on the role of school and family contexts in the sustainability of the changes observed in 24-hour behaviours, as their influence can be decisive in maintaining long-term effects. | Thanks for this comment. We added information to clarify this aspect. | Lines  401-407. |
| 46 | Finally, the section could be enriched with a more practical analysis of the findings, highlighting that reductions of between 1.5 and 2 hours per day in screen time are clinically relevant for adolescent health, even if the recommendation of two hours or less per day is not fully achieved. | Thanks for the comment. We have added to the discussion that the results are clinically relevant for adolescent health, even without full compliance with the ≤2-hour ST guideline. | Lines  366-374. |
| 47 | Finally, the section on limitations, strengths and suggestions for future studies should be restructured under the same heading. | Thanks for this suggestion. We have expanded this section. | Lines  424-437. |
|  | **Conclusion** | | |
| 48 | The conclusion summarises the findings well, highlighting improvements in sleep, reduced screen time and positive effects on some motivational constructs. However, the interpretation is limited, as it does not clarify that the effects were partial and that there were no significant improvements in physical activity or quality of life. It would be advisable to emphasise the practical value of modest changes in sleep and screen use, as well as to conclude with a clearer projection towards future research and application in school contexts. | Thanks for this suggestion. We have expanded this section. | Lines  454-461. |
|  | **References** | | |
| 49 | Please review the references section as they appear to be in a different font. | Thanks for the suggestion. We've corrected the font. | Lines 470. |
| 50 | Also review Figure 1, as it is of poor quality. | Thanks for the suggestion. The image quality and sharpness have improved. | Figure 1. |
