# Supplementary material for: Effects of a school-based intervention on 24-hour movement behaviors in adolescents: A quasi-experimental study
Source: PLoS One. 2025 Nov 21;20(11):e0336696. doi: 10.1371/journal.pone.0336696 (PMC12637913; doi:10.1371/journal.pone.0336696)
Supplement: Attachment — Submitted filename: Response to Reviewers.docx [file pone.0336696.s006.docx]

**Manuscript title: Effects of a school-based intervention on 24-hour movement behaviors in adolescents: A quasi-experimental study.**

Point-by-point response

| **Reviewer** | | |
| --- | --- | --- |
|  | ***Comment*** | ***Response*** |
| **#** | **Suggestions for improvement:** | |
| 1 | Clarification on support and control strategies in SDT  One of the most relevant points in this study is the use of SDT-based strategies. However, I would like to suggest clarifying whether the autonomy, competence, and relatedness strategies implemented in the intervention are primarily aimed at supporting these psychological needs or controlling them. According to SDT, these needs can be met in ways that either support or control individuals' motivation, and it is important to highlight this point in the article, as it could have implications for the interpretation of the results. This distinction is fundamental to understanding how the intervention impacts participants' motivation and behaviors. | Thanks for this comment. We edited Table 2 to indicate that all strategies were implemented to support the individuals' motivation. |
| 2 | Updating the tables  While I appreciate the author’s response to the reviewers' comments, I must point out that, for future occasions, it would be advisable to send a supplementary response to the reviewers' comments. The format in which the authors have responded to the comments makes it difficult to review the article, as the changes made to the manuscript are not clearly specified, nor are the exact sections that have been changed or added indicated. A more detailed and structured response would help reviewers to track the changes more efficiently, ensuring a smoother and more comprehensible review. | Thanks for this suggestion. We will consider it next submissions. |
| 3 | In Table 2 and other related tables, I suggest that the information be updated to better reflect the intervention strategies in relation to SDT. Specifically, it should be indicated whether the strategies applied are designed to promote an approach that supports psychological needs or whether there is any form of control over them. Including this information in the tables will provide greater clarity about the intervention design and its alignment with the proposed theory. | Seeing response to comment #1 |
